# Identifying personalized barriers for hypertension self-management from TASKS framework

**DOI:** 10.1186/s13104-024-06893-7

**Published:** 2024-08-14

**Authors:** Jiami Yang, Yong Zeng, Lin Yang, Nadia Khan, Shaminder Singh, Robin L. Walker, Rachel Eastwood, Hude Quan

**Affiliations:** 1 Department of Community Health Sciences, Faculty of Medicine, University of Calgary, Calgary, AB Canada; 2 Concordia Institute for Information Systems Engineering, Concordia University, Montreal, QC Canada; 3 Department of Cancer Epidemiology and Prevention Research, Cancer Care Alberta, Alberta Health Services, Calgary, AB Canada; 4 Departments of Oncology and Community Health Sciences, University of Calgary, Calgary, AB Canada; 5 Department of Medicine, Faculty of Medicine, University of British Columbia, Vancouver, BC Canada; 6 School of Nursing and Midwifery, Faculty of Health, Community and Education, Mount Royal University, Calgary, AB Canada

**Keywords:** Hypertension, Self-management, Personalized, Barriers, TASKS framework

## Abstract

**Objective:**

Effective management of hypertension requires not only medical intervention but also significant patient self-management. The challenge, however, lies in the diversity of patients' personal barriers to managing their condition. The objective of this research is to identify and categorize personalized barriers to hypertension self-management using the TASKS framework (Task, Affect, Skills, Knowledge, Stress). This study aims to enhance patient-centered strategies by aligning support with each patient's specific needs, recognizing the diversity in their unique circumstances, beliefs, emotional states, knowledge levels, and access to resources. This research is based on observations from a single study focused on eight patients, which may have been a part of a larger project.

**Results:**

The analysis of transcripts from eight patients and the Global Hypertension Practice Guidelines revealed 69 personalized barriers. These barriers were distributed as follows: emotional barriers (49%), knowledge barriers (24%), logical barriers (17%), and resource barriers (10%). The findings highlight the significant impact of emotional and knowledge-related challenges on hypertension self-management, including difficulties in home blood pressure monitoring and the use of monitoring tools. This study emphasizes the need for tailored interventions to address these prevalent barriers and improve hypertension management outcomes.

**Supplementary Information:**

The online version contains supplementary material available at 10.1186/s13104-024-06893-7.

## Introduction

Hypertension is a leading global health risk, significantly contributing to cardiovascular diseases such as stroke and heart failure and affecting mortality and morbidity rates worldwide [1–3]. Despite the effectiveness of lifestyle modifications and antihypertensive medications [4], patient adherence varies widely, with nonadherence rates between 10 and 80%, challenging the achievement of optimal blood pressure control [5, 6]. Self-management is critical in managing hypertension [7], requiring patients to take an active role in their health care, yet nearly 40% of patients discontinue crucial treatments, and over half fail to adhere to necessary behavioral changes [8]. Factors such as cultural beliefs and past healthcare experiences heavily influence patient attitudes toward self-management [9, 10].

Recognizing personalized barriers to hypertension self-management is essential for the successful implementation of interventions, aiming to bridge the evidence-to-practice gap in healthcare [11]. Personalized barrier identification allows for a deeper understanding of individual needs, preferences, and contextual factors, facilitating targeted interventions [12]. Traditional qualitative methods, like thematic analysis [13, 14], have been used to code interview transcripts in hypertension research, identifying common themes [15] across patient experiences. This method begins with interviews, letting themes emerge organically through deductive or inductive reasoning. Various frameworks like Consolidated Framework for Implementation Research (CFIR) [16], Theoretical Domains Framework (TDF) [17], Capability Opportunity Motivation Behavior (COM-B) [18], and Barriers and Facilitators in Implementation of Task-Sharing Mental Health Interventions (BeFITS-MH) [19] have provided predefined coding schemes. However, these methods often struggle to capture the diverse and personalized needs of patients [20].

To address these challenges, this study introduces the TASKS framework [12], which focuses on Task (T), Affect (A), Skills (S), Knowledge (K), and Stress (S), offering an approach to understanding the interplay between an individual's mental capabilities, external resources, and the demands of managing hypertension. The framework categorizes barriers into emotion, logic, knowledge, and resource-related, providing insights into the specific reasons behind patients' actions and decisions in self-managing hypertension. Originally applied in various fields such as education [21], engineering [22], sustainability [23] and beyond, the TASKS framework's adaptability presents a novel avenue for exploring personalized barriers in hypertension self-management. This research aims to evaluate the framework's effectiveness in identifying these barriers, marking a significant step towards enhancing patient-centered care and improving self-management outcomes in hypertension.

## Methods

### Study design and data information

This study employs the TASKS framework to identify personalized barriers from interview transcripts. Data were sourced from Global Hypertension Practice Guidelines [4] and anonymized interview transcripts from a prior study [13], with ethical clearance from the University of British Columbia's Clinical Research Ethics Board. Originally, nine patients from two focus groups were considered, but due to inefficiency in one patient's data, eight were ultimately analyzed.

Five transcript analyzers, comprising both medical and non-medical students, underwent intensive training on the coding process, which included defining the coding scheme and jointly coding 20 sentences. They then independently applied the TASKS framework to the transcripts of eight patients, resolving any coding discrepancies through discussion. The analyzers' agreement was assessed by independently coding two shared transcripts. This research aimed to validate the TASKS framework's utility in pinpointing personalized barriers to hypertension self-management.

### Coding hypertension guideline

We referred to the Global Hypertension Practice Guidelines [4] to identify the required TASK components: affect skills, knowledge (ASK), and resources necessary for specific workload/tasks (T). Workload, in this context, denotes the external load assigned by experts or governmental entities, such as recommendations made by physicians for patients. To break down this process, four key steps were undertaken: (1) extracting all required workloads specified in the Global Hypertension Practice Guidelines; (2) determining the life cycle associated with each workload [24]; (3) coding the ASK and resource requirements for each workload based on its life cycle; and (4) consolidating all stages of ASK and resource elements related to a particular workload.

### Identifying personalized barriers using the TASKS framework

#### Coding affect, skills, knowledge (ASK), and resource

In this step, we streamlined unstructured interview transcripts into a semi-structured format for detailed analysis. This involved classifying text by speaker and evaluating each sentence adhering to analyze underlying messages behind the interviewee's message including Affect (A), Skills (S), Knowledge (K) and Resource. Multiple analysts independently undertook this task to ensure a thorough examination of the data.

The TASKS framework differentiates between ASK and Resource. Affect relates to emotional experiences affecting task engagement, including attitudes, beliefs, feelings, and ethics. Skills involve cognitive and affective capabilities, emphasizing logical reasoning—deductive, inductive, abductive, and recursive [25] -to use knowledge in practical scenarios. Knowledge refers to understanding, including facts and cause-effect relationships related to the task at hand. Resources are considered as external aids like time, money, or physical tools.

For instance, in the provided transcript: "*My run marathons I've done 18 of them, I do yoga, I do everything that is possibly able to reduce blood pressure and has not been able to do that*," the patient exhibits (Affect) frustration and disappointment due to their extensive efforts not yielding the anticipated blood pressure reduction. They employ (Skills) deductive logic, assuming activities like running marathons and yoga would lower blood pressure based on common knowledge. This patient demonstrates (Knowledge) experience in activities linked to blood pressure reduction.

#### Evaluating and synthesizing coded ASK and resource

In the evaluation phase, an experienced TASKS framework analyst compares the conclusions drawn by multiple analysts, resolving conflicts and ensuring a definitive interpretation. These synthesized findings are combined to form a coherent result. During synthesis, the analyst categorizes and integrates the analyzed ASK and resource elements specific to each patient. The credibility of these findings is then verified by a hypertension specialist. This synthesis process provides a comprehensive view of patients' personas by encompassing their skills, knowledge, resources, and emotional and psychological factors. This approach offers a holistic understanding of their inner mental capabilities, defined as a composition of Affect, Skills, and Knowledge (ASK) according to the TASKS framework [26].

#### Barrier detection

The barrier detection employs a predictive approach, contrasting the requirements outlined in the Global Hypertension Practice Guidelines with individual patients' ASK (affect, skills, knowledge) and resources. For each workload, the analyst evaluates the ASK and resources to discern workload-specific barriers categorized to the TASKS framework (see Table 1). These workload-specific barriers are then grouped, forming a understanding of the barriers associated with each workload and individual patient. This detailed comprehension paves the way for crafting precise interventions and tailored support mechanisms, effectively addressing the identified barriers. This approach ensures that interventions are not generic but finely tuned to the unique self-management needs of each patient.

**Table 1:** Implementation barrier classification in the TASKS framework

Barriers	Content
Emotion barriers	Motivation, attitudes (such as cognitive/awareness, expectation, value), beliefs (such as acceptance, optimism), feelings (such as anxiety, pressure, fear), or ethics
Logic barriers	Thinking styles, thinking strategies, or reasoning methods
Knowledge barriers	The structure of knowledge, cognitive resources that are persons' past knowledge
Resource barriers	All environment components (such as time, money, and cognitive capacity)

## Results

### Hypertension guideline results

In our analysis, we systematically extracted and categorized all essential workloads outlined in the Global Hypertension Practice Guidelines [4] into four primary types: (1) Having a healthy lifestyle; (2) Monitoring blood pressure (BP) regularly at home, (3) Taking medication(s) regularly as prescribed, and 4) Creating a hypertension support system: family, friends, and healthcare professionals (HCPs). The comprehensive breakdown of necessary ASK and resources for each workload is detailed in Table 2. This table serves as a valuable implementation resource, aligning with the recommendations laid out in the Global Hypertension Practice Guidelines.

**Table 2:** Required ASK and resources for each workload in Global Hypertension Practice Guidelines

Workload	Affect	Skills	Knowledge	Resource
1. Having a healthy lifestyle Adhere to a balanced diet Restrict sodium intake and limit alcohol consumption Abstain from smoking and avoid environments where others smoke Engage in regular physical activity and maintain a healthy weight Strive for a stress-free lifestyle	1. Motivation to make the necessary effort in a healthy lifestyle 2. Patience in adhering to recommendations such as reducing sodium intake, limiting alcohol consumption, not smoking, and maintaining a healthy weight	Long-term thinking strategic Deduction logic Logical thinking Calculation Organization	3. Dietary Approaches to Stop Hypertension (DASH) diet and the importance of a balanced diet for managing hypertension 4. Limitations on sodium intake (alcohol intake, smoking) to control blood pressure 5. Healthy weight goals in relation to hypertension management 6. Different types of exercises are beneficial for managing hypertension 7. Knowledge about stress relaxation techniques	8. Friends and family 9. Time 10. Hypertension guidelines 11. DASH resources 12. Relaxation techniques 13. Take note of ways and health-related apps
2. Monitoring blood pressure (BP) regularly at home	1. Motivation to record daily readings 2. Willingness to confront their own BP readings 3. Patience with regular BP check-ups		4. Information about their BP 5. Knowledge of BP terminology and interpreting measurement 6. Realistic goals for hypertension level 7. Instructions for using the blood pressure monitor	8. Blood pressure monitor machine 9. Take note of ways 10. Time
3.Taking medication(s) regularly as prescribed	1. Motivation to take daily medications 2. Willingness to confront their own health conditions 3. Patience with consistently taking medications as prescribed 4. Trust in the effectiveness of medication or treatment		5. Professional knowledge regarding medication and prescribed information 6. Knowledge about side effects and adverse reactions	7. Medications
4. Creating a hypertension support system: family, friends, and healthcare professionals (HCPs) Regularly visit your HCP for checkups Seek immediate medical attention from your HCP in case of emergencies	1. Motivation to visit HCP for checkups 2. Patience with regularly visiting HCP for checkups 3. Trust in the physicians or HCPs 4. No white coat syndrome, which refers to elevated blood pressure in a medical setting due to anxiety or stress	5. Communication with others (friends and family, HCPs)	6. Information about their BP 7. Signs of side effects, such as stroke or heart attack	8. Friends and family 9. Physician/HCP 10. 911

### Barriers

Using the TASKS Framework, we compared the required Affect, Skills, and Knowledge (ASK) components outlined in the guidelines (Table 2) with each patient's individual ASK profile. Our analysis identified a new workload category, "2a. Using Blood Pressure Tools," emphasizing tool usage. Personalized barriers for eight patients were identified, each denoted by (). Supplementary file 1 provides more detailed patient-specific barriers information. We also categorized all barriers into emotion, logic, knowledge, and resource types, detailed in Table 3.

**Table 3:**
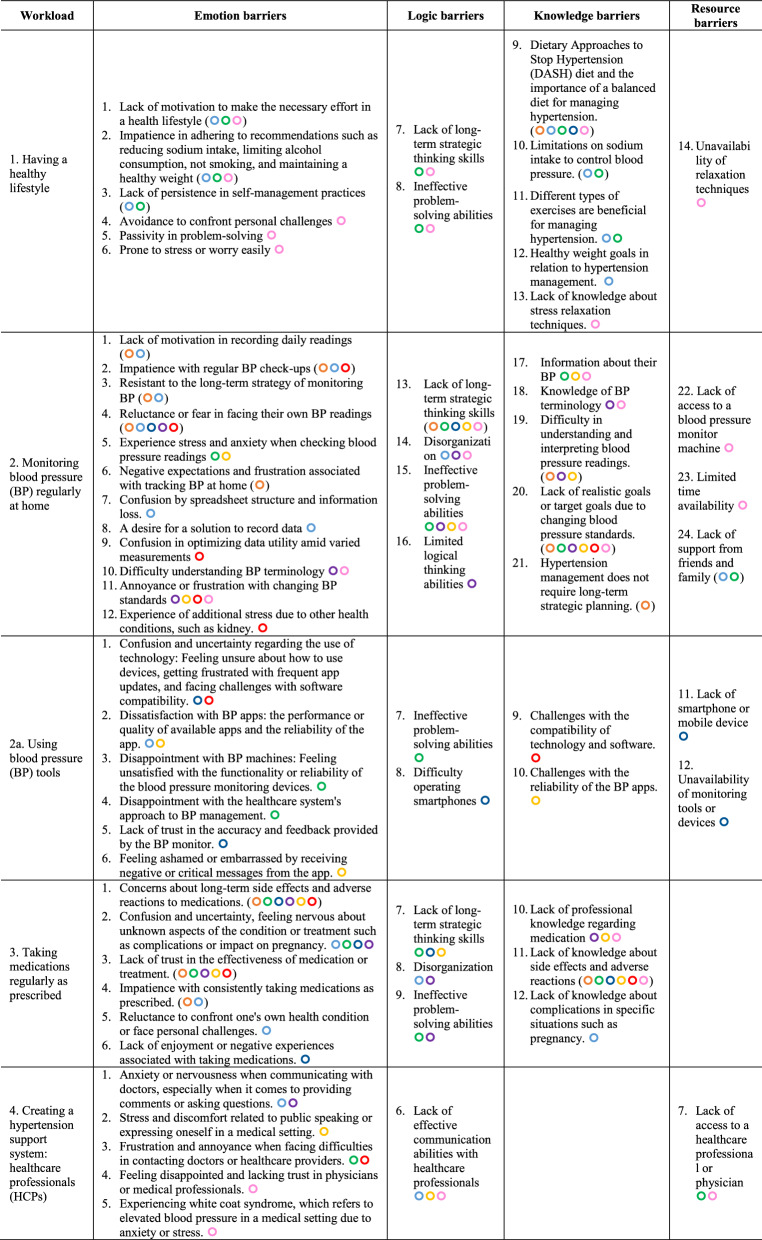
All hypertension self-management barriers using the TASKS framework

## Discussion

### What is the added value of personalized barriers for hypertension self-management?

Our research delved into personalized barriers in hypertension self-management, utilizing descriptive statistics to highlight common themes while acknowledging individual differences. Among the eight patients interviewed, a total of 69 barriers were identified, with emotion barriers being the most prevalent (49%), followed by knowledge (24%), logic (17%), and resource barriers (10%). Emotion barriers were the most prevalent, indicating significant stress and anxiety related to self-management tasks, such as monitoring blood pressure at home, which presented the highest challenge (34.78%). This was closely followed by difficulties in using blood pressure monitoring tools, medication management, and adopting a healthier lifestyle, each presenting substantial obstacles due to emotion and knowledge barriers. The least encountered barriers involved creating a support system with healthcare professionals (10.14%), yet still predominantly emotional.

By ranking these barriers (see Supplementary file 2), we aim to provide healthcare professionals with a clear understanding of the primary barriers faced by patients, guiding the development of targeted strategies to improve self-management outcomes. Determining the overall intervention approach and incorporating behavior change techniques have proven effective in altering behavior patterns within the target population [18, 27]. Emotional support, information provision, and enhancing patient-healthcare professional relationships emerge as key areas for intervention in hypertension management.

*Emotional support* Emotion barriers in hypertension self-management stem from fears and uncertainties about medication effects, as well as anxiety over blood pressure readings. Impatience and lack of motivation further hinder lifestyle changes and routine check-ups, creating a vicious cycle of stress and negative perception. Effective interventions foster trust and resilience. Cognitive-Behavioral Therapy (CBT) changes negative thought patterns and behaviors, and has been proven to positively impact hypertension outcomes, especially when group-based and long-term [28]. Mindfulness-Based Stress Reduction (MBSR) also helps manage stress and anxiety, improving outcomes in chronic disease management [29].

*Information provision* Understanding fluctuating blood pressure standards and medication side effects is challenging for patients. Personalized educational tools, available through digital platforms and brochures, will be essential. These resources offer clear insights into evolving standards and medication details, empowering patients to set realistic goals and manage potential side effects confidently. Encouraging peer-support groups and conducting regular knowledge assessments can further enhance understanding. By providing comprehensive, easy-to-understand information, patients can proactively navigate hypertension management, fostering a more informed and confident approach.

*Enhancing patient-healthcare professional relationships* Limited access to healthcare professionals is a significant hurdle. Motivational interviewing and shared decision-making, tailored to individual needs, can improve communication, boost engagement, and enhance self-efficacy [30, 31]. Telehealth services, such as community tele-paramedicine (CTP), and outreach programs further support patients by empowering them and transforming their journey from isolation to a sense of community, particularly for those in rural and remote areas [32–34]. Regular follow-ups and personalized communication also strengthen patient-provider relationships and improve adherence, highlighting the importance of empathetic and patient-centered care.

### Is the TASKS framework applicable for guiding data analysis?

**Fig. 1:**
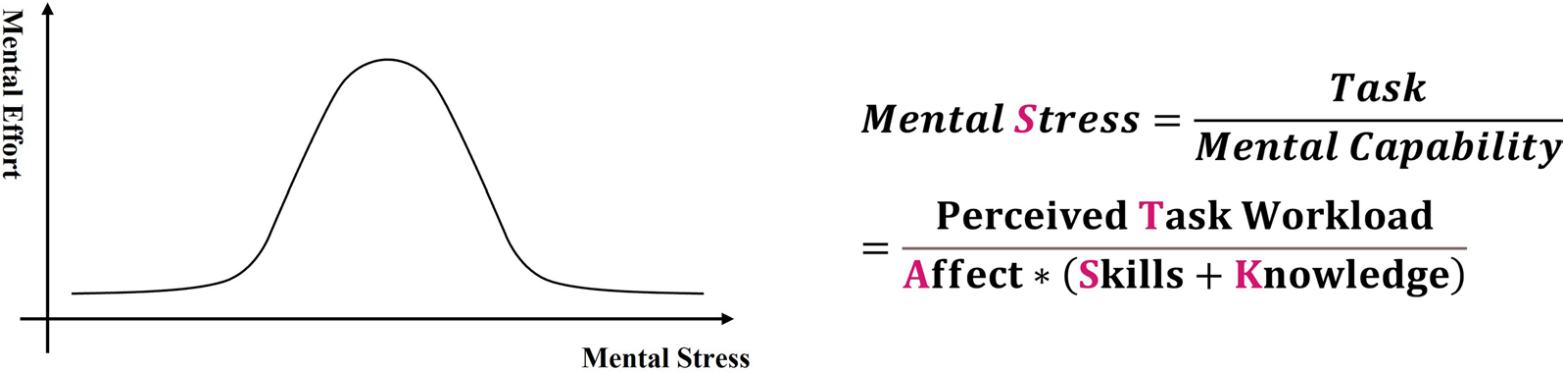
The relationship and information behind mental stress [12, 26]. (Left*:* an inverted U-shaped curve between mental stress and mental effort; Right: mental stress modeled by the ratio of perceived task workload to mental capability)

In health research, qualitative studies aim to comprehend the motivations and perceptions influencing health behaviors [35]. Employing a theoretical framework, like the TASKS framework, enhances the grounding of findings in robust theory, enriching the field's knowledge base. This framework uniquely focuses on the complex interplay between an individual's tasks and their mental capabilities—Affect, Skills, and Knowledge—and how this interplay is affected by mental stress, following an inverted U-shaped curve [36]. Patient’s performance relies on their mental effort, which depends on their mental stress. This dynamic demonstrates how mental effort correlates with mental stress, wherein both low and high stress levels can diminish mental effort, but moderate stress may optimize it [12] (see Fig. 1 left).

The TASKS framework categorizes implementation barriers into emotion, logic, knowledge, and resource types through a precise equation involving the ratio of perceived tasks workload to mental capability [26] (see Fig. 1 right). It systematically aligns guideline requirements with individual circumstances through a top-down to bottom-up method for coding and modeling mental capabilities (ASK) and resources. This process identifies hypertension self-management barriers by comparing guideline requirements with personal situations, both structured by ASK and resources. This alignment naturally extends the four types of barriers into disease- and patient-specific corresponding barriers, such as emotional responses (e.g., concerns about long-term medication effects), logical barriers (e.g., ineffective communication with healthcare providers), knowledge gaps (e.g., lack of necessary medication knowledge), and resource limitations (e.g., insufficient tools or support). Integrating this data enables a comprehensive analysis and supports tailored interventions.

Furthermore, the framework explains the interactive relationship between the perception of workload and the application of skills and knowledge. It underscores the significance of understanding emotional responses to perceived workloads, thereby establishing a recursive logic in behavioral performance [25]. Achieving a balance between workload and mental capability is essential [37], underscoring the need for an in-depth analysis of the cause-and-effect relationships among various barriers [38]. Such detailed analysis can uncover valuable insights, enabling the development of targeted intervention strategies that meet the unique needs of patients, ultimately improving self-management outcomes.

### Limitation and future works

Our study, focusing solely on hypertension self-management barriers, may not apply to other disease contexts, suggesting the need to test the TASKS framework more broadly. With a limited sample of eight patients, findings might not capture the full diversity of self-management experiences; thus, a larger sample is recommended for greater reliability. Moreover, conducting interviews only in English could introduce cultural biases and exclude non-English speakers.

Future research should include multiple languages or translation services to address linguistic and cultural differences in self-management barriers. A key direction is developing tools to streamline the analysis of personalized barriers. While the TASKS framework is effective, it is time-consuming and labor-intensive. Integrating the TASKS framework with technologies like natural language processing (NLP) and large language models can create automated or semi-automated tools, reducing subjective judgment and enhancing scalability and efficiency in personalized healthcare research. This advancement could significantly improve personalized healthcare, making it more accessible and effective for a broader range of diseases. Additionally, research should explore the impact of various intervention techniques for different barriers, such as cognitive-behavioral therapy and motivational interviewing, and expand the TASKS framework's application in diverse healthcare settings.

## Conclusion

In conclusion, our study highlights the critical importance of personalized barriers in the self-management of hypertension, with emotion and knowledge barriers identified as the most significant. By applying the TASKS framework, we have unraveled the interplay between individual mental capabilities and the demands of self-managing hypertension. Emotion barriers were the most significant, followed by knowledge, logic, and resource barriers, emphasizing the need for tailored interventions. The TASKS framework guided our data analysis, effectively categorizing barriers and facilitating the development of precise interventions. While our focus was on hypertension, the framework's adaptability suggests its broader applicability in healthcare research. Nonetheless, limitations such as a small sample size and linguistic bias warrant further investigation. Overall, our research contributes to promoting patient-centered care and refining hypertension management strategies.

### Supplementary Information

Supplementary Material 1.

Supplementary Material 2.

## Data Availability

The datasets used during the current study are available from the Prof. Nadia Khan upon reasonable request. Analyzed data is provided within the supplementary information files.
