# Supplementary material for: Identifying personalized barriers for hypertension self-management from TASKS framework
Source: BMC Res Notes. 2024 Aug 14;17:224. doi: 10.1186/s13104-024-06893-7 (PMC11323669; doi:10.1186/s13104-024-06893-7)
Supplement: Supplementary file 1 — Supplementary Material 1. [file 13104_2024_6893_MOESM1_ESM.docx]

**Personalized Barriers for Hypertension Self-Management**

# A (🞆)

| ***A*** |  |
| --- | --- |
| **Emotion barriers** | **Monitoring BP**:   - Lack of motivation in recording daily readings - Negative expectations and frustration associated with tracking BP at home - Impatience with regular BP check-ups - Reluctance or fear in facing their own BP readings - Resistant to the long-term strategy of monitoring BP   **Taking Medications**:   - Concerns about long-term side effects and adverse reactions to medications - Lack of trust in the effectiveness of medication or treatment - Impatience with consistently taking medications as prescribed |
| **Knowledge barriers** | **Healthy Lifestyle**:   - DASH diet and the importance of a balanced diet for managing hypertension.   **Monitoring BP**:   - Hypertension management does not require long-term strategic planning. - Difficulty in understanding and interpreting blood pressure readings. - Lack of realistic goals or target goals due to changing blood pressure standards.   **Taking Medication**: Lack of professional knowledge regarding medication, including understanding side effects |
| **Logic barriers** | **Monitoring BP:** Lack of long-term strategic thinking skills |
| **Resource barriers** | **None** |

# B (🞆)

| ***B*** | |
| --- | --- |
| **Emotion barriers** | **Healthy Lifestyle**: Lack of motivation to make the necessary effort in a healthy lifestyle, Impatience in adhering to recommendations such as reducing sodium intake, limiting alcohol consumption, not smoking, and maintaining a healthy weight; Lack of persistence in self-management practices  **Monitoring BP**: Reluctance or fear in facing their own BP readings; confusion by spreadsheet structure and information loss; Lack of motivation in recording daily readings, Impatience with regular BP check-ups; Resistant to the long-term strategy of monitoring BP. a desire for a solution to record data  **BP tools:** Dissatisfaction with BP apps: Feeling let down by the performance or quality of available apps.  **Medications**: Confusion and uncertainty, feeling nervous about unknown aspects of the condition or treatment such as complication or impact on pregnancy; Reluctance to confront one's own health condition or face personal challenges; Impatience with consistently taking medications as prescribed.  **Communication with HCP**: Anxiety or nervousness when communicating with doctors, especially when it comes to providing comments or asking questions. |
| **Knowledge barriers** | **Healthy Lifestyle**: DASH diet and the importance of a balanced diet for managing hypertension, Limitations on sodium intake to control blood pressure; Different types of exercises that are beneficial for managing hypertension, Healthy weight goals in relation to hypertension management.  **Medication**: Lack of professional knowledge regarding medication, including understanding side effects, and complications in specific situations such as pregnancy. |
| **Logic barriers** | Lack of effective communication abilities with healthcare professionals, disorganization |
| **Resource barriers** | **Monitoring BP**: Lack of support from friends and family (maybe) |

# C (🞆)

| ***C*** | |
| --- | --- |
| **Emotion barriers** | **Healthy Lifestyle**: Lack of motivation to make the necessary effort in a healthy lifestyle, Impatience in adhering to recommendations such as reducing sodium intake, limiting alcohol consumption, not smoking, and maintaining a healthy weight, physically active, and maintaining a healthy weight; Lack of persistence in self-management practices  **Monitoring BP**: Experience of stress and anxiety when checking blood pressure readings  **BP tools:** disappointing for BP machine/ healthcare system  **Medications**: Concerns about long-term side effects and adverse reactions to medications, Confusion and uncertainty, feeling nervous about unknown aspects of the condition or treatment, Lack of trust in the effectiveness of medication or treatment  **Exception**: confused uncertainty  **HCP**: Frustration and annoyance when facing difficulties in contacting doctors or healthcare providers. |
| **Knowledge barriers** | **Healthy Lifestyle**: DASH diet and the importance of a balanced diet for managing hypertension, Limitations on sodium intake to control blood pressure, different types of exercise that are beneficial for managing hypertension.  **Monitoring BP**: information about BP, Lack of realistic goals or target goals due to changing blood pressure standards.  **Medication**: professional knowledge for medication, side effects for medication |
| **Logic barriers** | Problem-solving, long-term thinking strategic |
| **Resource barriers** | **Monitoring BP**: friends and family  physician/HCP |

# D (🞆)

| ***D*** | |
| --- | --- |
| **Emotion barriers** | **Monitoring BP**: Reluctance or fear in facing their own BP readings  **BP tool**: Confusion and uncertainty regarding the use of technology, Lack of trust in the accuracy and feedback provided by the BP monitor.  **Medications**: Concerns about long-term side effects and adverse reactions to medications, Confusion and uncertainty, feeling nervous about unknown aspects of the condition or treatment such as complication or impact on pregnancy, Lack of enjoyment or negative experiences associated with taking medications. |
| **Knowledge barriers** | **Healthy Lifestyle**: DASH diet and the importance of a balanced diet for managing hypertension.  **Medications**: Lack of professional knowledge regarding side effects of medication |
| **Logic barriers** | Long-term thinking strategic  **BP tools**: Difficulty operating smartphones |
| **Resource barriers** | **Healthy Lifestyle**: weight monitor tools  **BP tools**: lack of smartphone |

# E (🞆)

| ***E*** | |
| --- | --- |
| **Emotion barriers** | **Monitoring BP**: Difficulty understanding BP terminology, Annoyance or frustration with changing BP standards (specific targets of BP level), Reluctance or fear in facing their own BP readings  **Medications**: Concerns about long-term side effects and adverse reactions to medications; Confusion and uncertainty, feeling nervous about unknown aspects of the condition or treatment; Lack of trust in the effectiveness of medication or treatment,  **HCP**: scared doctors, cautious around clinicians |
| **Knowledge barriers** | **Monitoring BP:** Lack of realistic goals or target goals due to changing blood pressure standards; Knowledge about BP terminology; Difficulty in understanding and interpreting blood pressure readings  **Medications:** professional knowledge of medication |
| **Logic barriers** | Organization, problem solving, logical thinking |
| **Resource barriers** | None |

# F (🞆)

| ***F*** | |
| --- | --- |
| **Emotion barriers** | **Monitoring BP**: Annoyance or frustration with changing BP standards, Experience of stress and anxiety when checking blood pressure readings  **BP tools**: Dissatisfaction with BP apps (reliability of app)  Feeling ashamed or embarrassed by receiving negative or critical messages from the app  **Medications**: Concerns about long-term side effects and adverse reactions to medications, Lack of trust in the effectiveness of medication or treatment  **Communication with HCP**: stressful public speaking  **Himself**: dare to face themselves, worry, frustrated uncertainty |
| **Knowledge barriers** | **Monitoring BP**: Information about their BP; Lack of realistic goals or target goals due to changing blood pressure standards, Difficulty in understanding and interpreting blood pressure readings  **BP tools**: Challenges with the reliability of the BP apps  **Medication**: lack of professional knowledge of medication, lack of knowledge about side effects of medication |
| **Logic barriers** | long-term thinking strategic, problem-solving  communication |
| **Resource barriers** | None |

# G (🞆)

| ***G*** | |
| --- | --- |
| **Emotion barriers** | **Monitoring BP**: stressful for the kidney; Confusion in optimizing data utility amid varied measurements; Annoyance or frustration with changing BP standards; Reluctance or fear in facing their own BP readings; Impatience with regular BP check-ups  **BP tools**: Confusion and uncertainty regarding the use of technology: Feeling unsure about how to use devices, getting frustrated with frequent app updates, and facing challenges with software compatibility.  **Medications**: Concerns about long-term side effects and adverse reactions to medications; Lack of trust in the effectiveness of medication or treatment  **HCP**: Frustration and annoyance when facing difficulties in contacting doctors or healthcare providers. |
| **Knowledge barriers** | **Monitoring BP**: Lack of realistic goals or target goals due to changing blood pressure standards.  **BP tools**: Challenges with the compatibility of technology and software  **Medications**: Lack of knowledge about side effects and adverse reactions |
| **Logic barriers** | None |
| **Resource barriers** | None |

# H (🞆)

| ***H*** | |
| --- | --- |
| **Emotion barriers** | **Healthy Lifestyle**: Lack of motivation to make the necessary effort in health lifestyle; Impatience in adhering to recommendations such as reducing sodium intake, limiting alcohol consumption, not smoking, and maintaining a healthy weight, persistence; Avoidance to confront personal challenges; Passivity in problem-solving; Prone to stress or worry easily  **Monitoring BP**: Annoyance or frustration with changing BP standards, Difficulty understanding BP terminology  **HCP**: Feeling disappointed and lacking trust in doctors or medical professionals; Experiencing white coat syndrome, which refers to elevated blood pressure in a medical setting due to anxiety or stress. |
| **Knowledge barriers** | **Healthy Lifestyle**: DASH diet and the importance of a balanced diet for managing hypertension; Limitations on sodium intake to control blood pressure; Information about stress relaxation techniques  **Monitoring BP**: Information about their BP; Knowledge about BP terminology Lack of realistic goals or target goals due to changing blood pressure standards.  **Medications**: Lack of professional knowledge regarding medication, Lack of knowledge about side effects and adverse reactions |
| **Logic barriers** | Long-term thinking strategic  Organization, analysts, problem-solving  **HCP:** communication with HCP |
| **Resource barriers** | **Healthy Lifestyle**: relaxation techniques, therapist about stress  **Monitoring BP**: BP machine, time  **HCP:** physician |
