# Supplementary material for: Identifying personalized barriers for hypertension self-management from TASKS framework
Source: BMC Res Notes. 2024 Aug 14;17:224. doi: 10.1186/s13104-024-06893-7 (PMC11323669; doi:10.1186/s13104-024-06893-7)
Supplement: Supplementary file 2 — Supplementary Material 2. [file 13104_2024_6893_MOESM2_ESM.docx]

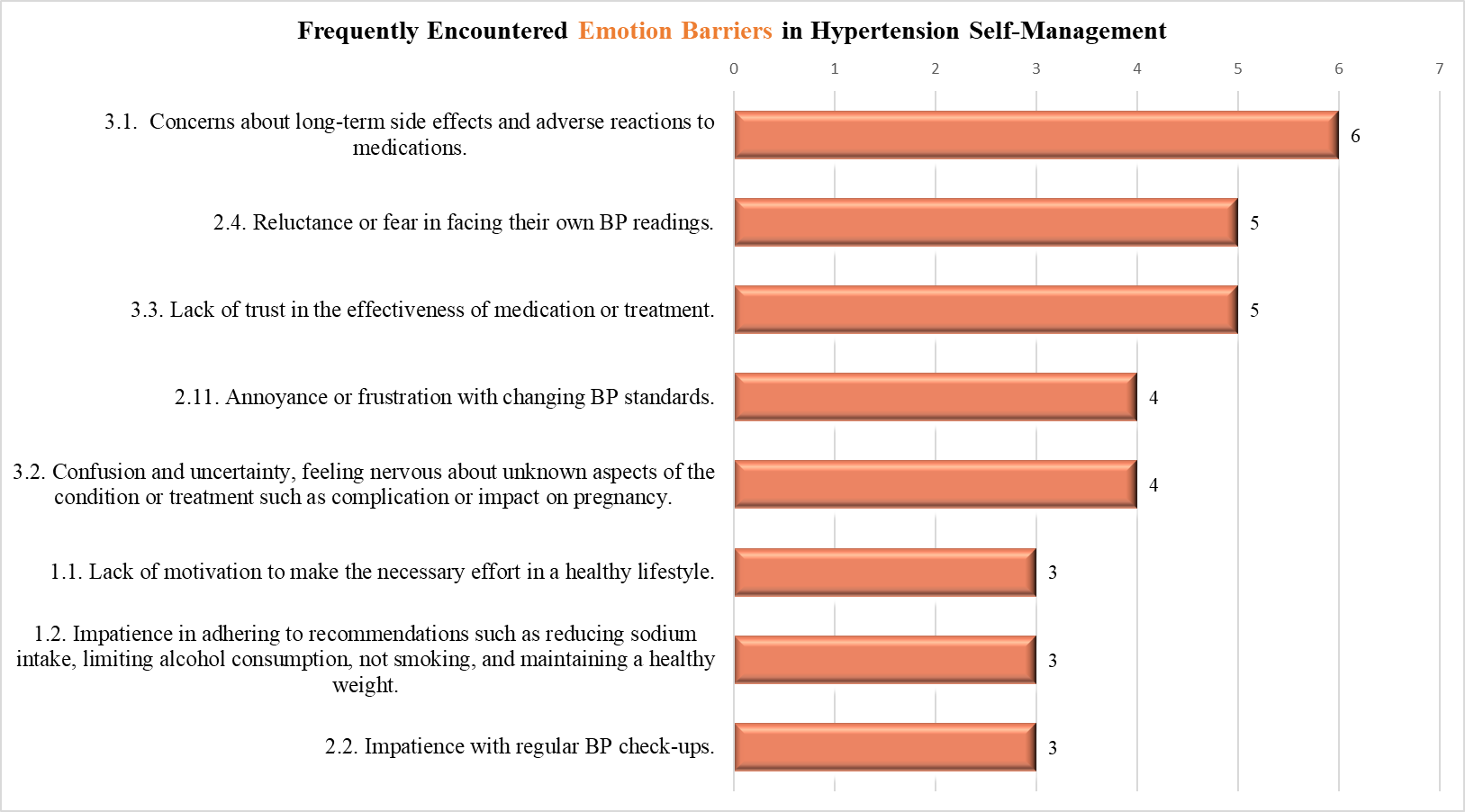


A) Emotion barriers B) Logic barriers

C) Knowledge barriers D) Resource barriers

Figure S1. Frequently encountered four types of barriers in hypertension self-management (*BP:* Blood pressure, *HCPs:* healthcare professionals, *DASH:* Dietary Approaches to Stop Hypertension)
